# Amniotic band syndrome in a monochorionic diamniotic twin pregnancy after rupture of the dividing membrane in the early second trimester: A case report

**DOI:** 10.1186/s12884-021-03948-6

**Published:** 2021-06-28

**Authors:** Mizuki Nakashima, Takashi Iizuka, Kyosuke Kagami, Rena Yamazaki, Masanori Ono, Hiroshi Fujiwara

**Affiliations:** Department of Obstetrics and Gynecology, Graduate School of Medical Science, Kanazawa University, Takaramachi 13-1, Kanazawa, Ishikawa 920-8641 Japan

**Keywords:** Twins, Amniotic band syndrome, Prenatal ultrasonography, Prenatal diagnosis, Monochorionic diamniotic

## Abstract

**Background:**

Amniotic band syndrome is a rare phenomenon, but it can result in serious complications. We report herein our experience of amniotic band syndrome in a monochorionic diamniotic twin pregnancy where rupture of the dividing membrane occurred early in the second trimester.

**Case Presentation:**

A 29-year-old nulliparous woman was referred to us for management of her monochorionic diamniotic twin pregnancy at 10 weeks of gestation. When we were unable to identify a dividing membrane at 15 weeks of gestation using two-dimensional ultrasonography, we used three-dimensional ultrasonography to confirm its absence. Both modalities showed that the left arm of baby B was swollen and attached to a membranous structure originating from the placenta at 18 weeks of gestation. Tangled umbilical cords were noted on magnetic resonance imaging at 18 weeks of gestation. Emergency cesarean delivery was performed at 30 weeks of gestation because of the nonreassuring fetal status of baby A. The left arm of baby B had a constrictive ring with a skin defect. Both neonates had an uncomplicated postnatal course and were discharged around 2 months after delivery.

**Conclusions:**

Attention should be paid to the potential for amniotic band syndrome if rupture of the dividing membrane between twins is noted during early gestation.

## Background

Amniotic band syndrome (ABS) is a rare condition that can result in deformation, constriction, and even amputation of fetal parts. The condition results when fetal parts are trapped by amniotic bands, which are fibrous strands formed when the amniotic sac ruptures or tears [1]. Iatrogenic ABS, which is also referred to as pseudo-amniotic band syndrome, is caused by medical procedures such as fetoscopic laser photocoagulation [2] and septostomy [3], both used for treating twin-to-twin transfusion syndrome (TTTS). There are also reports of pseudo-amniotic band syndrome caused by amniocentesis [4, 5]. Theoretically, spontaneous septostomy of the dividing membrane in a twin pregnancy could also cause ABS, but only a single case has been reported [6]. Septostomy of a dividing membrane is reportedly related to increased fetal mortality due to the resulting transition from a monochorionic diamniotic (MCDA) gestation to a monochorionic monoamniotic (MCMA) gestation [7]. Cord entanglement and true-knot formation are serious perinatal complications associated with monoamniotic gestations, including those that result from septostomy [8–10]. We report a rare case of ABS in a MCDA twin pregnancy that occurred after rupture of the dividing membrane in the early second trimester.

## Case Presentation

**Fig. 1:**
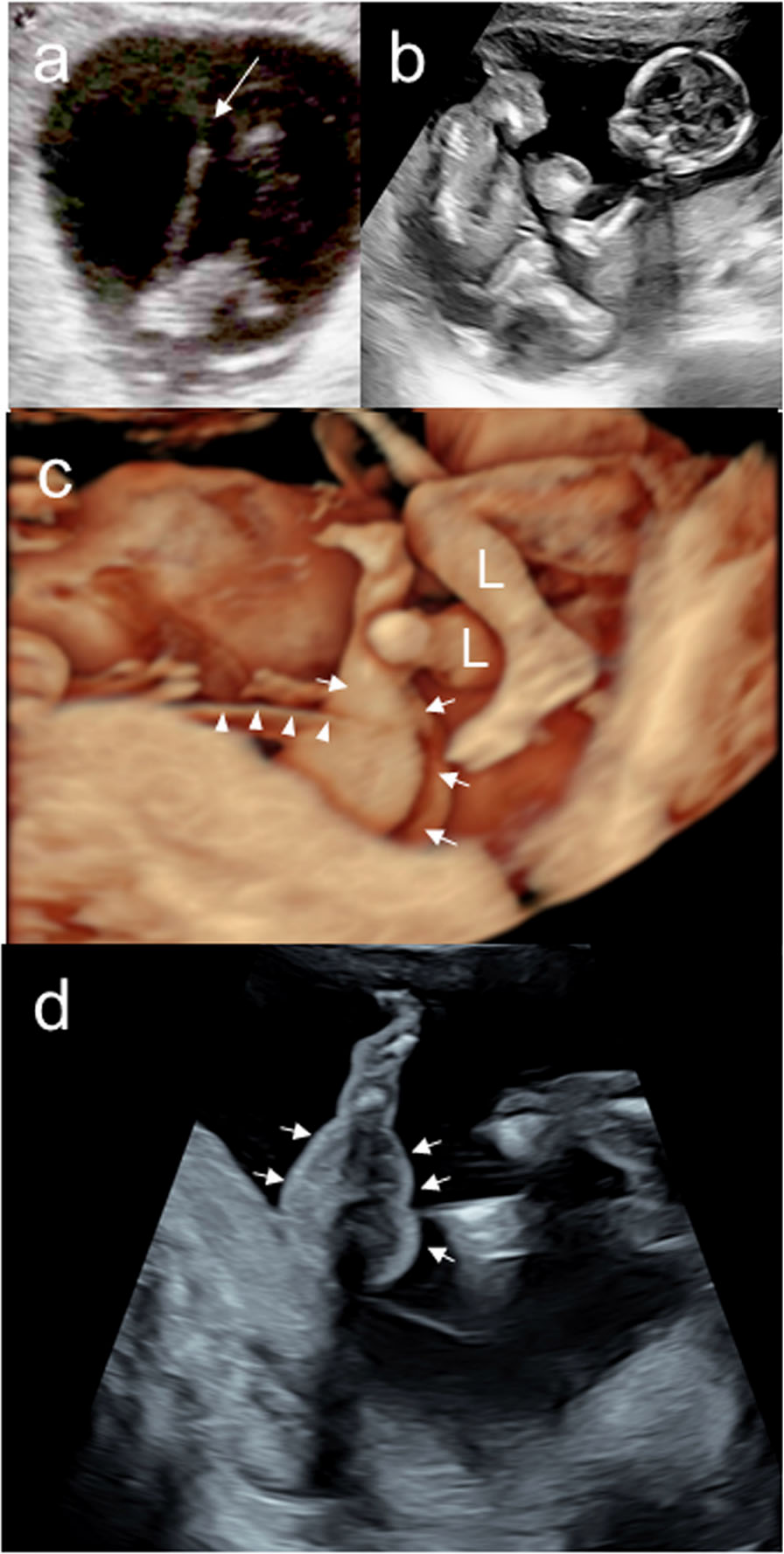
Fetal ultrasonography. **a** Transvaginal ultrasonography at 8 weeks of gestation. The lack of the “twin peak” sign, also called the lambda sign (arrow), indicates an MCDA twin pregnancy. **b** Transabdominal fetal ultrasonography at 15 weeks of gestation. There is no evidence of a dividing membrane. **c** Transabdominal three-dimensional ultrasonography at 18 weeks of gestation. Baby B has a swollen upper left arm (arrows), and part of the amniotic membrane (arrowheads) is attached to this arm. *L* legs of baby A, *P* placenta. **d** Transabdominal two-dimensional ultrasonography at 18 weeks of gestation. Baby B has a swollen upper left arm (arrows)

**Fig. 2:**
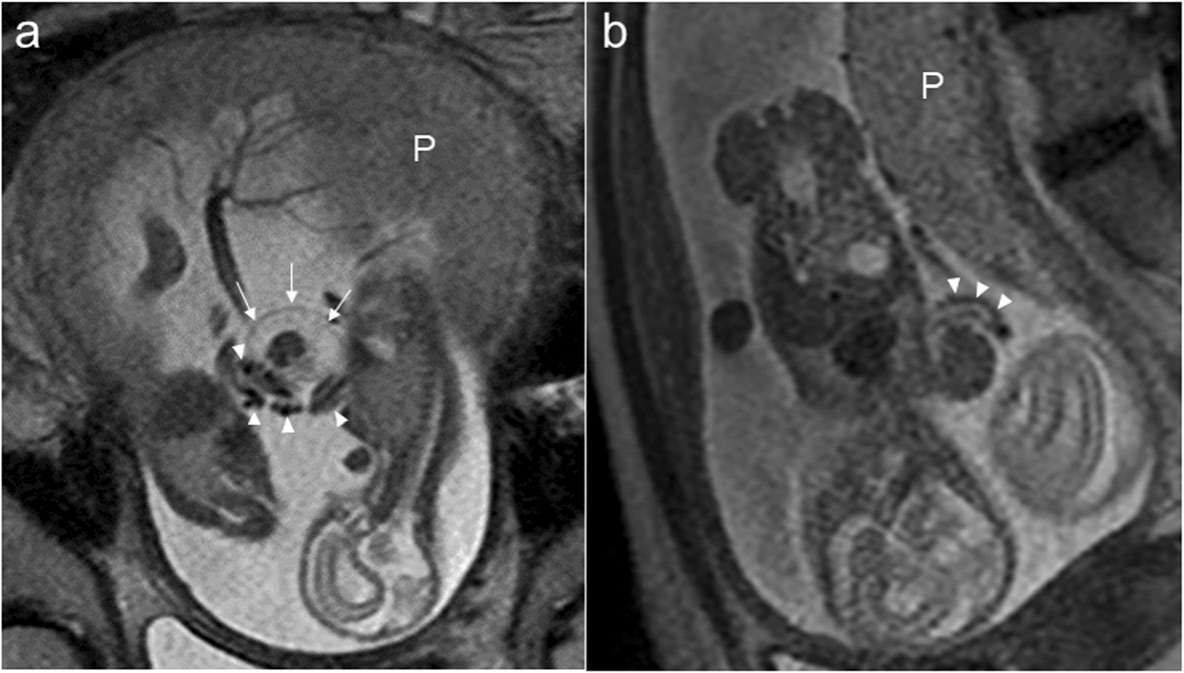
Magnetic resonance imaging. Magnetic resonance imaging at 18 weeks of gestation. The upper left arm of baby B is swollen, and edema is present (**a**, arrows). The umbilical cord is seen encircling the same arm (**a**, **b**, arrowheads)

A 29-year-old nulliparous woman achieved pregnancy after the administration of human chorionic gonadotropin to trigger ovulation. She was diagnosed with an MCDA twin pregnancy at 8 weeks of gestation (Fig. 1a) and referred to our hospital for management at 10 weeks of gestation. At 13 weeks of gestation, we confirmed the presence of a dividing membrane between the twins. However, at 15 weeks of gestation, a large portion of the dividing membrane was missing on ultrasonography (Fig. 1b), leading us to suspect rupture of the dividing membrane between 13 and 15 weeks. After the diagnosis of suspected spontaneous septostomy at 15 weeks, we performed careful weekly follow-up. At 18 weeks of gestation, we performed three-dimensional ultrasonography and noted that baby B had a swollen upper left arm (Fig. 1c, arrows) and that a membranous structure originating from the placenta was attached to the swollen upper left arm (Fig. 1c, arrowheads). We noted that the swollen upper left arm was actually in the same cavity as baby A (Fig. 1c). Baby A was anatomically normal. Two umbilical cord insertion sites were noted on the placenta—one on either side of the partial dividing membrane. Next, we used magnetic resonance imaging (MRI) to reassess the amniotic membranes, umbilical cords, and fetal morphology. MRI confirmed that baby B’s upper left arm was swollen and that baby B’s umbilical cord was encircling the arm (Fig. 2a and b). We suspected that the umbilical cord had become entrapped by the amniotic band.

We kept the patient under close follow-up and subsequently noted mild growth restriction in both babies, but there was no sign of TTTS or selective fetal growth restriction. At 25 weeks of gestation, MRI showed a reduction in the swelling of baby B’s upper left arm and there was no visible amniotic band attached to baby B’s arm. Cesarean delivery was scheduled for 34 weeks of gestation.

**Fig. 3:**
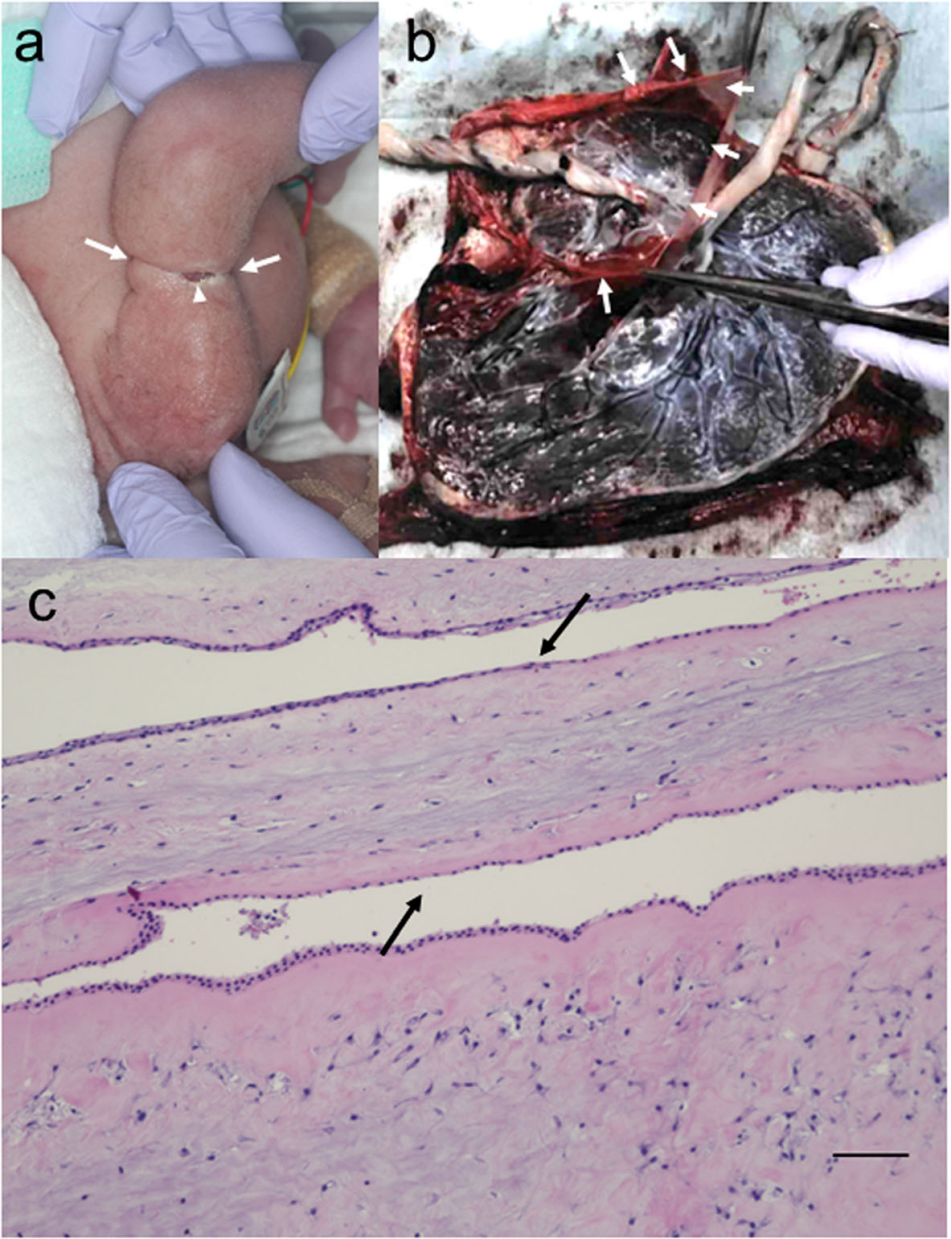
Postnatal findings: Baby B and placenta. **a** The left arm of baby B shows a constrictive ring (arrows) with a 1-cm skin defect (arrowhead). The amniotic bands are unidentifiable. **b** Gross examination of the placenta. A part of the dividing membrane is identifiable (arrows), but most of it is missing. **c** Histopathological examination of the amniotic membranes. There is a double amnion layer (arrows) without a chorion. Scale bar: 100 μm

We performed electronic fetal heart rate monitoring 3 times daily. While the fetal heart rate was nonreassuring at 30 weeks, there was no abnormality in the middle cerebral artery or ductus venosus Doppler studies, nor was there an abnormal amniotic fluid volume. We therefore suspected that the nonreassuring pattern was due to umbilical cord entanglement. Our intraoperative findings confirmed the presence of entangled umbilical cords. Baby A had a birth weight of 1285 g and Apgar scores of 8 and 9 at 1 and 5 minutes, respectively. Baby B weighed 1323 g and had Apgar scores of 7 and 8, respectively. Both twins were small for their gestational age. The left arm of baby B was functionally normal but showed a constrictive ring with a skin defect (Fig. 3a); there were no identifiable amniotic bands. Most of the amniotic membrane separating the babies was missing on gross examination of the placenta (Fig. 3b). The remaining membrane was confirmed to be consistent with an MCDA gestation on microscopic examination (Fig. 3c).

The maternal postoperative course was uneventful, and the patient was discharged 1 week after delivery. Both neonates followed an uneventful postnatal course. Baby B’s skin defect epithelialized and formed a scar at around 2 months of age. Both infants were discharged 64 days after delivery.

## Discussion and Conclusion

We report herein a patient with an MCDA twin pregnancy with ABS noted in one of the twins. To the best of our knowledge, only one previous case of ABS after spontaneous rupture of the dividing membrane in MCDA twins has been reported [6]. However, this previous case report did not describe any antenatal ultrasound findings of ABS, nor did it describe the status of the dividing membrane during the pregnancy. Here, we were able to observe in-utero ABS after early second-trimester rupture of the dividing membrane.

**Fig. 4:**
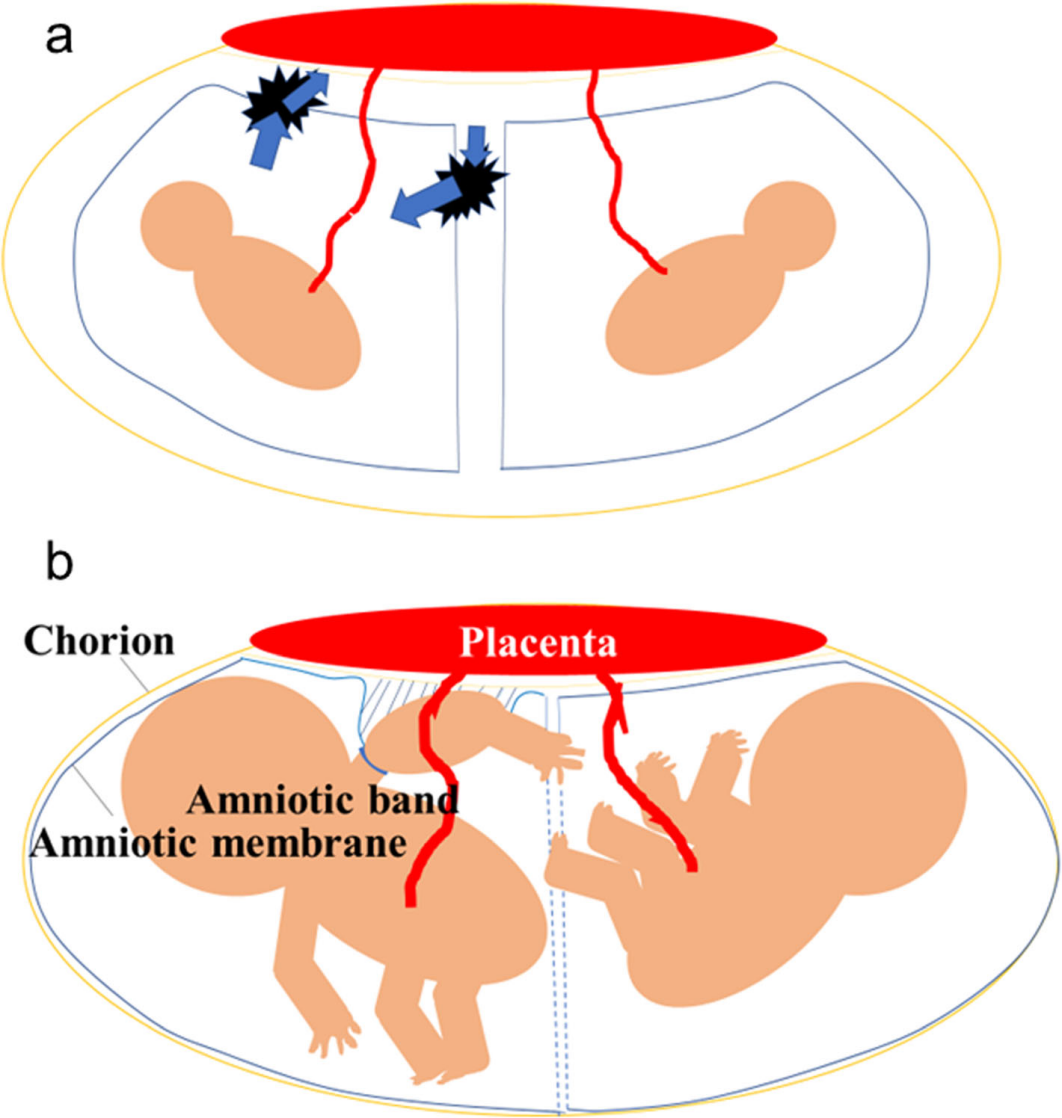
Predicted pathology of amniotic band syndrome following rupture of the dividing membrane. **a** Rupture of the amniotic membrane allows inflow of amniotic fluid and expansion of the space between the amniotic membrane and chorion. **b** Baby B had a swollen upper left arm and the membranous structure originating from the placenta was attached to the swollen upper left arm. The swollen upper left arm was in the same cavity as baby A. This figure was depicted by authors using PowerPoint®

At 12 to 15 weeks, the period during which the placenta is formed, there is a space between the placenta and amniotic membrane. In our patient, the presence of a dividing membrane was confirmed at 13 weeks of gestation, but the dividing membrane could not be seen at 15 weeks of gestation: there was an amniotic membrane rupture between 13 and 15 weeks. As shown in Fig. 4a and b, rupture of the amniotic membrane allows inflow of amniotic fluid and expansion of the space between the amniotic membrane and chorion. Baby B’s upper arm may have entered this newly expanded space. Indeed, we noted a swollen upper arm between the placenta and amniotic membrane on three-dimensional ultrasonography, which showed that the swollen upper arm was actually in the same cavity as baby A. We suggest that baby B’s upper arm pierced the amniotic membrane at another site, causing ABS. We want to emphasize that attention should be paid to the potential for ABS if rupture of the dividing membrane between twins is noted during early gestation, when the chorion and amnion have not yet fused.

Two theories of pathogenesis have been suggested for ABS: exogenous and endogenous theories. The exogenous theory was first described by Torpin [1] and states that early partial rupture of the amniotic membrane allows parts of the fetal body to enter the space between the amniotic membrane and chorion and they get entrapped by fibrous strands representing amniotic bands. The endogenous theory is endorsed by Streeter, who explains that ABS is caused by developmental abnormalities of the amniotic cavity [11]. Several reports show that ABS is more frequent in monozygotic twin than in dizygotic twin pregnancies [12, 13]. This case was originally MCDA twins and was considered to be prone to develop abnormalities of the amniotic cavity. In the early second trimester, we detected amniotic membrane rupture. Since the amniotic rupture was close to the dividing membrane, the dividing membrane also ruptured creating a single amniotic cavity despite the presence of MCDA twins.

There are case reports describing fetoscopic surgery in patients with ABS, with the intervention conferring a good prognosis [14, 15]. However, fetoscopic surgery can cause complications such as uterine bleeding and preterm rupture of membranes. We conducted frequent ultrasonography, which showed improvement in the swollen upper arm of baby B. We interpreted this as a release of the ABS and therefore did not perform fetoscopic surgery.

A large part of the dividing membrane in this twin gestation was missing at the time of delivery (Fig. 3b). Considering that baby B’s swollen arm improved between 18 and 25 weeks of gestation, the amniotic band that initially entrapped the fetal arm may have been released at some point. We suggest that the dividing membrane in this MCDA twin gestation spontaneously ruptured in the early second trimester. One high-risk maternal-fetal medicine unit reports that the rate of spontaneous rupture is as high as 1.8% [16]. Rupture of the dividing membrane causes a condition equivalent to a monoamniotic twin pregnancy, which is associated with a higher risk of intrauterine fetal demise than diamniotic twin pregnancies, due to the possibility of umbilical cord entanglement [17]. In our patient, there was also the possibility that baby B’s umbilical cord was initially entrapped by amniotic bands. Because we followed our patient closely, with careful attention to umbilical cord blood flow and fetal development, she was able to successfully deliver live newborn twins.

A diagnosis of ABS can be made with two-dimensional ultrasound, but three-dimensional ultrasonography allows for the observation of amniotic bands and any related fetal abnormalities [18, 19]. The addition of three-dimensional ultrasonography is useful for assessing fetal abnormalities [20]. In this case, three-dimensional ultrasonography was able to visualize the swollen fetal arm that was entrapped by an amniotic band. Fetal MRI can also be used to visualize amniotic bands [21], although this modality is more useful in later pregnancy, when overlapping fetal parts make it difficult to observe details of anatomy on ultrasound. Fetal MRI enables a wide range of images and provides objective evaluation, regardless of the skill of the examiner. In this case, we observed entangled umbilical cords that were free of entrapment by amniotic bands; we were not able to see these features on ultrasonography.

In conclusion, attention should be paid to the potential for ABS if rupture of the dividing membrane between twins is noted during early gestation.

## Data Availability

Not applicable.

## Abbreviations

ABS: Amniotic band syndrome
MCDA: Monochorionic diamniotic
MCDA: Monochorionic monoamniotic
MRI: Magnetic resonance imaging
TTTS: Twin-to-twin transfusion syndrome
